# Structure and Properties of High-Strength Ti Grade 4 Prepared by Severe Plastic Deformation and Subsequent Heat Treatment

**DOI:** 10.3390/ma13051116

**Published:** 2020-03-03

**Authors:** Alena Michalcová, Dalibor Vojtěch, Jaroslav Vavřík, Kristína Bartha, Přemysl Beran, Jan Drahokoupil, Jan Džugan, Jan Palán, Jakub Čížek, Pavel Lejček

**Affiliations:** 1 Department of Metals and Corrosion Engineering, University of Chemistry and Technology in Prague, Technická 5, 166 28 Prague 6, Czech Republic; vojtechd@vscht.cz (D.V.); vavrika@vscht.cz (J.V.); 2 Department of Physics of Materials, Charles University, Ke Karlovu 5, 121 16 Prague, Czech Republic; kristina.vaclavova@gmail.com; 3 Nuclear Physics Institute ASCR in Řež, Husinec-Řež 130, 250 68 Řež, Czech Republic; pberan@ujf.cas.cz; 4 European Spallation Source ERIC, 222 70 Lund, Sweden; 5 Institute of Physics, Academy of Sciences of the Czech Republic, Na Slovance 2, 182 21 Prague 8, Czech Republic; JanDrahokoupil@seznam.cz (J.D.); lejcekp@fzu.cz (P.L.); 6 COMTES FHT, Department of Mechanical Testing and Thermophysical Measurement, Průmyslová 995, 334 41 Dobřany, Czech Republic; jdzugan@comtesfht.cz (J.D.); jan.palan@comtesfht.cz (J.P.); 7 Department of Low-Temperature Physics, Faculty of Mathematics and Physics, Charles University, V Holešovičkách 2, CZ-180 00 Prague 8, Czech Republic; jakub.cizek@mff.cuni.cz

**Keywords:** Ti grade 4, conform SPD, XRD, neutron diffraction, TEM, PAS

## Abstract

Severe plastic deformation represented by three passes in Conform SPD and subsequent rotary swaging was applied on Ti grade 4. This process caused extreme strengthening of material, accompanied by reduction of ductility. Mechanical properties of such material were then tuned by a suitable heat treatment. Measurements of in situ electrical resistance, in situ XRD and hardness indicated the appropriate temperature to be 450 °C for the heat treatment required to obtain desired mechanical properties. The optimal duration of annealing was stated to be 3 h. As was verified by neutron diffraction, SEM and TEM microstructure observation, the material underwent recrystallization during this heat treatment. That was documented by changes of the grain shape and evaluation of crystallite size, as well as of the reduction of internal stresses. In annealed state, the yield stress and ultimate tensile stress decreased form 1205 to 871 MPa and 1224 to 950 MPa, respectively, while the ductility increased from 7.8% to 25.1%. This study also shows that mechanical properties of Ti grade 4 processed by continual industrially applicable process (Conform SPD) are comparable with those obtained by ECAP.

## 1. Introduction

Titanium and its alloys exhibit frequent utilization in a wide range of application fields, from aircraft industry [1,2,3] to medicine [2,3,4]. Nowadays, titanium with commercial purity has been very popular for structural applications [4,5,6,7]. The main reason is that the impurities present in commercially pure titanium (mainly oxygen and iron) do not limit the possibility of medical application, unlike some other potentially harmful alloying elements. Commercially pure titanium is differentiated on the basis of the amount of impurities by grades, where grade 1 means the lowest content of impurities. Among titanium with different commercial purities, one of the most studied is Ti grade 4, with maximal allowed content of O 0.4% and maximal allowed content of Fe 0.5% [8].

It is possible to strengthen Ti grade 4 by severe plastic deformation (SPD). One of the powerful processes of SPD is the technology Conform SPD developed at COMTES FHT, Czech Republic. The Conform SPD involves severe plastic deformation. It is essentially equivalent to the CONFORM ECAP method, but it has a narrower channel angle of 90°, which provides more severe deformation. The main advantage of these methods is that they allow continuous production [9]. The combination of Conform SPD with rotary swaging was also found to be very effective [10,11]. The Conform SPD process can be in simplification described as continuous (and because of this industrially applicable) variation on equal channel angular pressing (ECAP), which is well described technology, combined with conform process (continuous extrusion). It can be expected that the dependence of microstructure and mechanical properties on processing parameters in Conform SPD is analogous to those observed for ECAP. The values of the yield stress (YS), ultimate tensile stress (UTS) and elongation to fracture (A) depend on the number of passes of ECAP (N) and processing temperature (T), as listed in Table 1.

The values presented in Table 1 are a compilation of the literature results showing that YS and UTS increase with an increasing number of ECAP passes. It is also obvious that the first three passes have the significant role for strengthening, while the further passes lead to a negligible increase of YS and UTS values, if at all. By subsequent plastic deformation, represented by drawing or rotary swaging, both YS and UTS soared. This is a sign of deformation strengthening, because in the same time, the ductility value slumped.

It was described by Průša et al. [5] that hardness increased from 170.4 to 330.6 HV1 after three passes by Conform SPD, followed by rotary swaging. The ductility decreases with increasing strength, which worsens workability of the material. Possible solving of this problem is to apply subsequent heat treatment after severe plastic deformation, as it was described for Ti grade 2 [9]. Suitable heat treatment in this case leads to release of internal stresses but does not cause grain coarsening. 

As a result, the ductility is improved to plausible values, and the Hall-Petch strengthening provided by fine grains is preserved. Table 2 [13] gives the values of the selected mechanical properties of Ti grade 4 after severe plastic deformation and subsequent heat treatment. Both YS and UTS increased after four passes in ECAP, and ductility decreased. After short-term annealing (15 min), YS and UTS reached even higher values. The explanation might be connected with slight rotation of deformed crystals, thus forming a texture in the material [13]. The value of elongation-to-fracture did not change as compared to the ECAPed material. Longer annealing times caused a decrease of YS and UTS but an increase of ductility.

The aim of our research was to find suitable heat treatment for Ti grade 4 after severe plastic deformation by Conform SPD and rotary swaging that produced a high strength (YS and UTS) of material and proved an increase of ductility. The temperatures and durations of the heat treatments are selected to be acceptably low and short for possible industrial application. 

## 2. Materials and Methods 

Titanium with commercial purity, Ti grade 4, was processed via 3 passes by Conform SPD, at 200 °C, followed by rotary swaging with diameter reduction of 80%. The final sample (3 Conform SPD + RS) was a rod of the diameter of 5 mm.

The electrical resistance was measured in situ, during heating, using a standard four-point method. The voltage and electrical current were measured simultaneously by nanovoltmeter Keithley 2182 and SourceMeter Keithley 2400 devices (Cleveland, OH, USA) respectively. The measurement took place in a specially designed furnace which allows a precise heating of the sample in an inert Ar atmosphere. The principle of this method is precisely described elsewhere [14,15]. The electrical resistance was measured during linear heating from room temperature, up to 800 °C, with a heating rate of 5 °C/min.

Structure evolution during heat treatment was studied by in situ XRD at room temperature, 50 °C, 100 °C, 150 °C and from 200 °C up to 540 °C, with a step of 20 °C, heating speed of 1 K/s and measuring time for each point of 12 min. Isothermal annealing was also performed at 450 °C for 246 min, with a step of 13 min. The measurements were performed by using PANanalytical X´Pert PRO equipped by high speed X’Celerator detector and by Anton Paar HTK 2000 heating chamber (Almelo, The Netherlands) enabling.

The electrical resistance and in situ XRD measurements were correlated with microhardness measurement HV 0.1 after annealing for 1 h in the temperature range 400–700 °C, with a step of 25 °C. The microhardness was also measured after annealing at 450 °C with a step of 15 min.

Neutron powder diffraction was employed to study structure evolution in the whole sample. The diffraction experiment was performed on selected samples, i.e., initial material, material annealed at 450, 475, 500 and 525 °C for 1 h and at 450 °C for 3 h. The diffraction experiment was done using the MEREDIT diffractometer (Řež, Czech Republic) at the Nuclear Physics Institute ASCR in Řež, the Czech Republic. A neutron beam with the wavelength of 1.46 Å (mosaic copper monochromator) was used to irradiate samples placed in vanadium containers with a diameter of 13 mm. To minimize the influence of the sample texture, the containers were rotating along the vertical axis during the measurement. Data were collected at room temperature in a 2θ range of 4°–144° with a step size of 0.08°. XRD and neutron diffraction data were processed by Rietveld structural refinement, to get the information about crystallite size and about internal stresses in case of XRD.

The microstructure of the selected samples was observed by SEM (TESCAN VEGA 3 LMU, equipped by Oxford instruments EDS detector) and by TEM (Jeol 2200 FS, equipped by Oxford instruments EDS detector) (Tokyo, Japan). TEM samples were prepared by mechanical grinding, dimpling and ion polishing, using Gatan PIPs (Pleasanton, CA, USA).

The lifetime of positrons on Ti grade 4 was measured by positron annihilation spectroscopy (PAS, Canberra, Australia). The ^22^NaCl radioactive source deposited on a 2 µm thick mylar foil was used and placed between two identical samples of the studied material. Positron lifetime was measured by a digital spectrometer, with a time resolution of 145 ps (FWHM ^22^Na) [16]. 

Microtensile testing was performed, using a LabControl machine (Ostrava, Czech Republic). The samples were dog-bone shaped, with a length of 4.5 mm, width of 1 mm and thickness of 0.5 mm.

## 3. Results and Discussion

**Figure 1:**
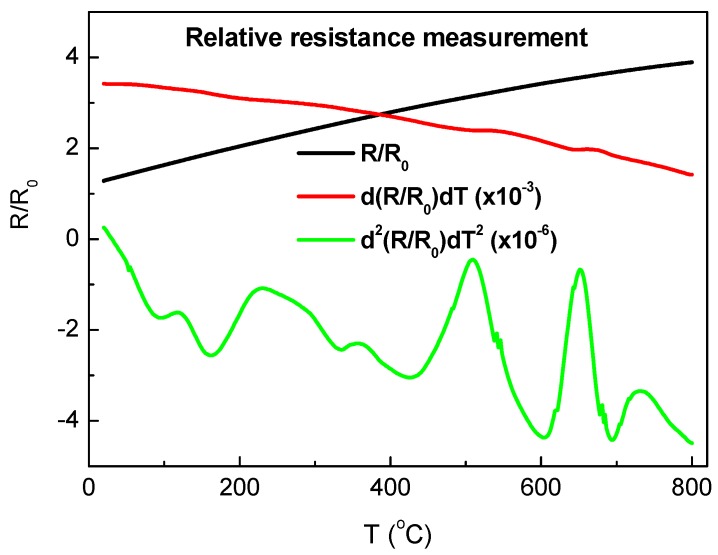
Relative resistance variations of 3 Conform SPD + RS Ti during heating: linear temperature dependence, the first derivative of relative resistance and the second derivative of relative resistance.

Figure 1 shows the temperature dependence of the electrical resistance of Ti grade 4 after three passes of Conform SPD and rotary swaging (3 Conform SPD + RS). The relative resistance R/R_0_, where R is the electric resistance measured at the given temperature (T) and R_0_ is the electric resistance at room temperature, is plotted at the vertical axis. During linear heating up to 800 °C the electrical resistance increased almost three times, as documented in Figure 1. Up to approximately 125 °C, the curve is stabilizing. Several small peaks are visible at higher temperature at the first derivate curve (red in Figure 1). To highlight them, the second derivation is plotted in green in Figure 1. Based on the assumption that these peaks correspond to undergoing microstructural changes, the temperature of 450 °C was chosen for subsequent heating and microstructural observations. Other peaks were observed at temperatures of approximately 600 and 700 °C, and these temperatures are not suitable for heat treatment, as they would lead to rapid grain coarsening and decrease of strength of materials.

**Figure 2:**
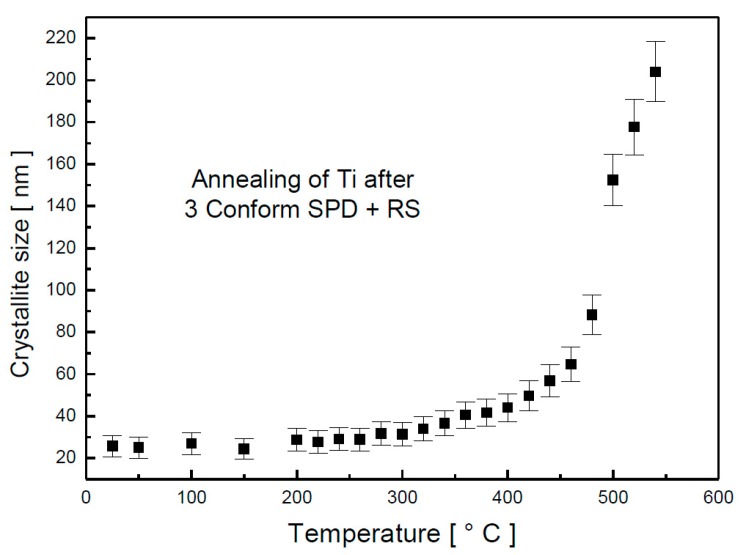
Crystallite size dependence on temperature determined by in situ XRD.

No changes in phase composition were observed by in situ XRD during annealing, but significant changes in crystallite size took place, as shown in Figure 2. The crystallite size was almost constant up to 300 °C with the value of approximately 30 nm. Between 300 and 440 °C, the crystallite size slightly increased. Above 460 °C, the soar of the crystallite size was significant, and at 540 °C, the crystallite size reached the value of approximately 200 nm.

**Figure 3:**
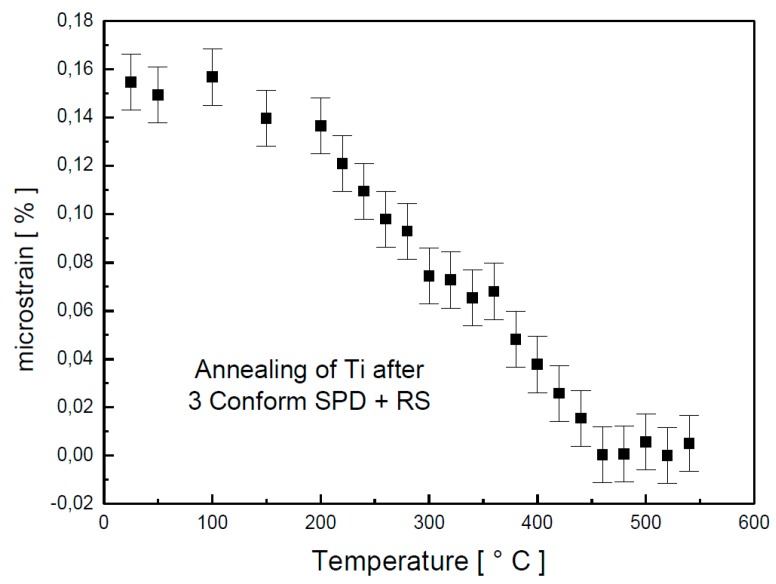
Microstrain dependence on temperature determined by in situ XRD.

The dependence of the microstrain on annealing temperature is plotted in Figure 3. Up to 200 °C, the microstrain decreased slowly. However, above this temperature, the decrease was rapid so that, at 460 °C, all microstrains in the material were relaxed. As the major changes happened around the temperature of 450 °C, isothermal annealing, accompanied with in situ XRD, was performed. 

**Figure 4:**
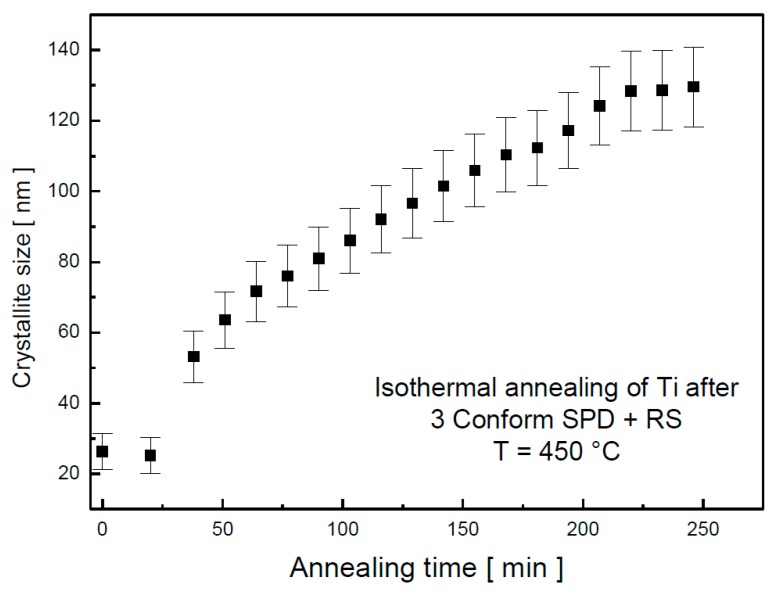
XRD Crystallite size dependence on annealing time at 450 °C determined by in situ XRD.

The crystallite size evolution is shown in Figure 4. Its values were slightly increasing up to 200 min. Further annealing at this temperature did not seem to affect the crystallite size.

**Figure 5:**
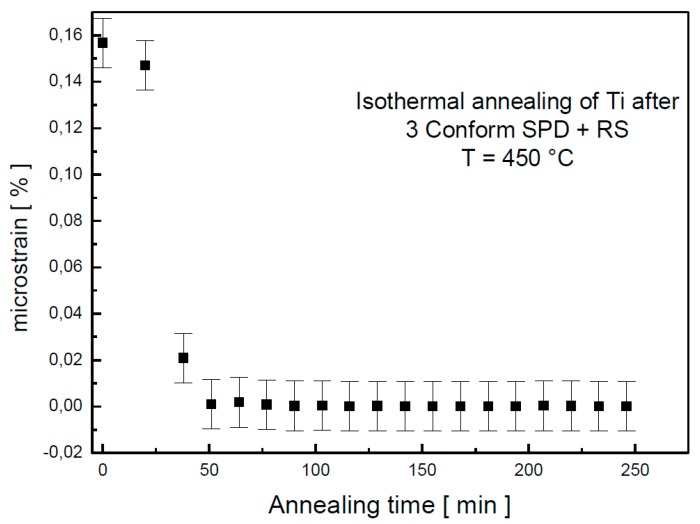
Microstrain dependence on annealing time at 450 °C determined by in situ XRD.

**Figure 6:**
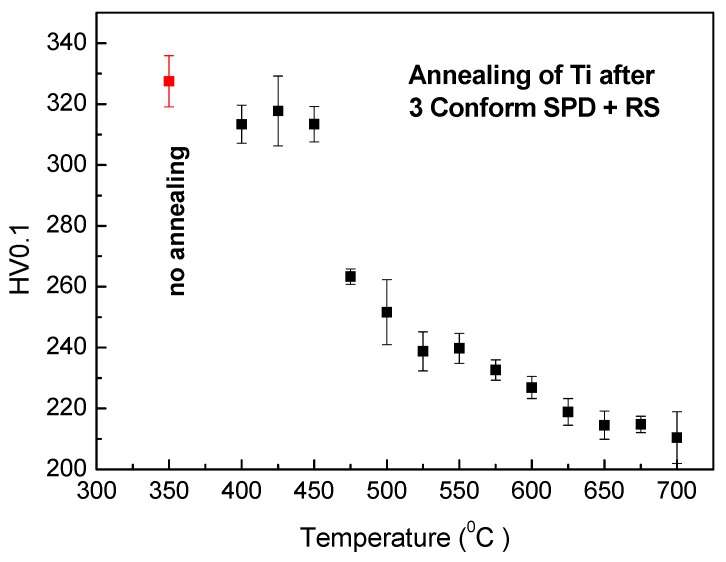
Hardness dependence on annealing temperature for samples annealed for 1 h.

The relaxation of microstrain happened around 50 min of annealing, as documented in Figure 5. The observations from in situ XRD and electrical resistance measurements were completed by microhardness measurement after annealing. The values of the microhardness after 1 h of annealing are plotted against annealing temperature in Figure 6. Although the first two methods represent in situ measurements while the hardness was tested on a cold sample after annealing, all these results are in a good agreement. A slight decrease of the microhardness was observed under 450 °C, which could be cause by release of internal stresses accumulated in material during severe plastic deformation. A significant change happens in the material after 1 h annealing at 475 °C, because at this temperature, recrystallization starts. Above this temperature, the microhardness slightly decreases; this decrease is probably connected with grain coarsening.

**Figure 7:**
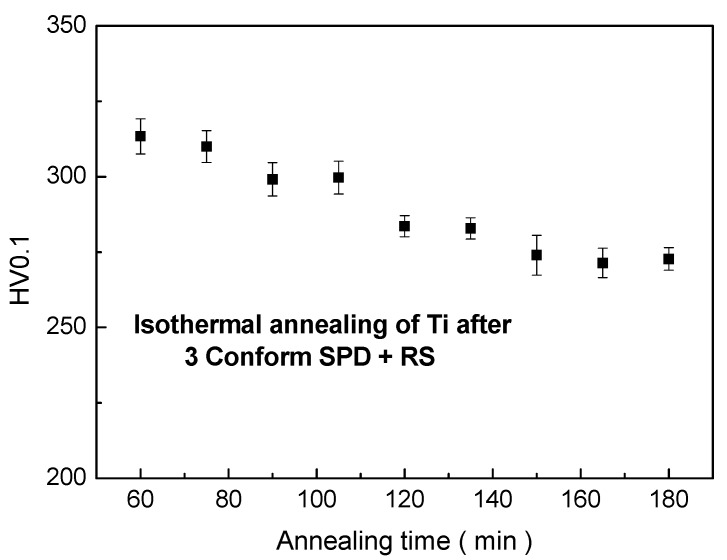
Hardness dependence on annealing time at 450 °C.

During annealing at 450 °C, a moderate decrease of the microhardness values was observed with increasing annealing time, as documented in Figure 7. This observation is in agreement with the increase of crystallite size, documented in Figure 4.

**Figure 8:**
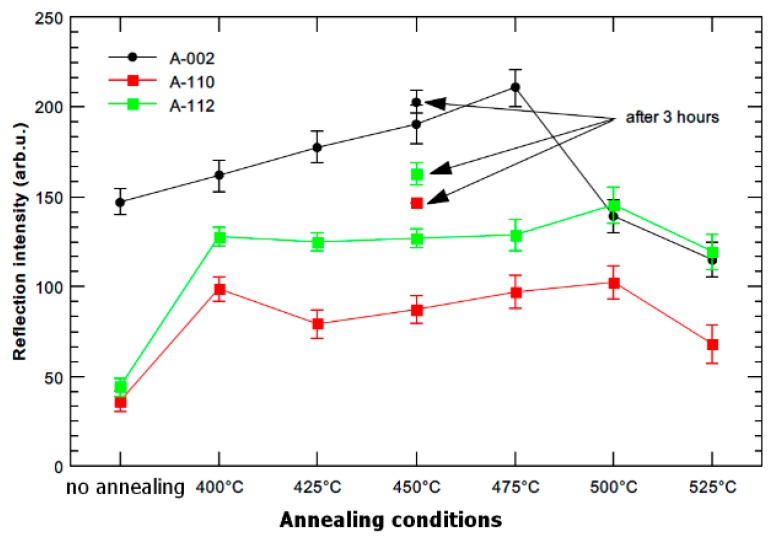
Reflection intensity dependence on annealing temperature for samples annealed at 400–525 °C for 1 h and the sample annealed at 450 °C for 3 h (neutron powder diffraction).

Selected samples were studied by neutron powder diffraction, to characterize changes in the whole material and not just a surface layer, as it is in XRD. Figure 8 shows the evolution of reflection intensity. The non-annealed sample (3 Conform SPD + RS) is textured along the long axes of the rod. This orientation, which is typical for ECAPed samples [12,13], is strengthened by the effect of rotary swaging. The results in Figure 8 are in good agreement with observation done by SAED [13]. The intensity of the 002 reflection increased with increasing annealing temperatures (and time for 450 °C) up to 475 °C. It implies that lattice rotation and a texture characteristic changed during the annealing. The decrease of intensity of the 002 reflection above 475 °C indicates the recrystallization of material.

**Figure 9:**
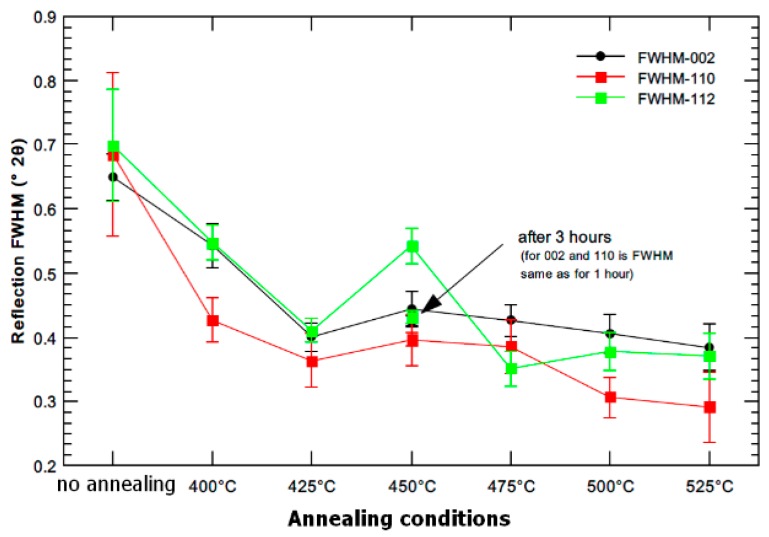
FWHM dependence on annealing temperature for samples annealed at 400–525 °C for 1 h and the sample annealed for 3 h at 450 °C (neutron powder diffraction).

Broadening of the diffraction peak is presented in Figure 9 as full width in a half maxima (FWHM). These values are influenced by grain size and by internal stresses in the sample. The initial material (3 Conform SPD + RS) contains very fine crystallites and significant internal stresses (microstrain of 0.15% determined from XRD, as shown in Figure 3 and Figure 5). Relaxation of material is demonstrated by decrease of FWHM values up to 425 °C. This observation proves the XRD results presented in Figure 2.

**Figure 10:**
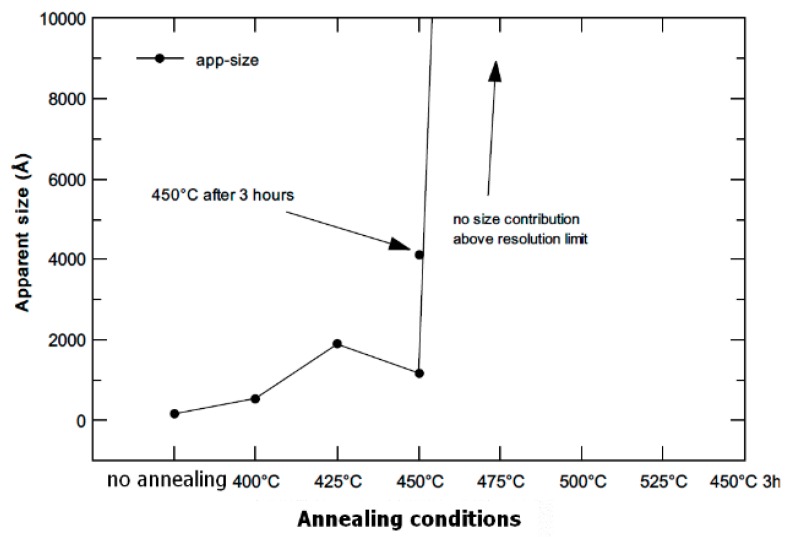
Apparent size (crystallite size) dependence on annealing temperature for samples annealed at 400–450 °C for 1 h and for sample annealed for 3 h at 450 °C (neutron powder diffraction).

Similar to XRD, the crystallite size (in this case denoted as apparent size) was determined from FWHM valued, as illustrated in Figure 10. The observed trend is identical, although the absolute numbers are different. It is caused by the difference in the nature of each radiation (XRD, neutrons). The XRD is more sensitive to the surface changes which occur at elevated temperatures, while the neutrons bring the information from the whole sample volume. The observed trend is identical, although the absolute numbers are different. It is caused by the difference in the nature of each radiation (XRD, neutrons). The XRD is more sensitive to the surface changes which occur at elevated temperature, whilst the neutrons bring the information from the whole sample volume. The size of the coherent domains (scattering parts of the material) can be in the absolute values different between surface and volume, which is what can lead to the mentioned discrepancy.

**Figure 11:**
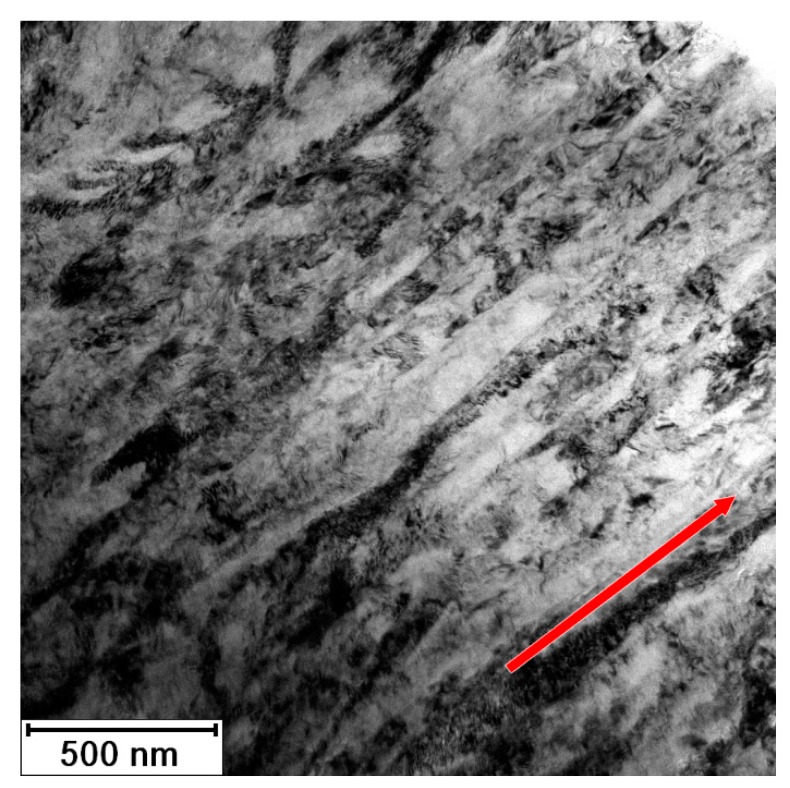
Microstructure of Ti grade 4 after 3 Conform SPD + RS; arrow shows RS direction (TEM).

Figure 11 documents the microstructure of initial material (3 Conform SPD + RS). The material consists of fine elongated grains oriented parallel to the long axis of the sample (identical to the direction of processing). The width of grains is less than 100 nm (minimal Feret’s diameter is 80 ± 30 nm) and the length exceeds 1 µm (maximal Feret’s diameter is 1200 ± 400 nm). This grain size indicates that the coherently diffracting domains observed by both diffraction methods corresponds to the subgrains (with the size of 20 nm). The material contains inherent elastic and plastic anisotropy, which was proven by electron micrographs. The accumulated dislocations form the dark regions in the TEM micrograph in Figure 11. According to EDS elemental mapping the impurities (Fe and O) rich regions were not found in this sample. There are two possible explanations, (i) the impurities are distributed homogenously and their content is under the detection limit of EDS; or (ii) the field of view in the TEM that is in range of few micrometers per sample, is limited. In case that the impurities form large objects inhomogenously distributed in the material, they might not be contained in the electron transparent part of TEM sample.

**Figure 12:**
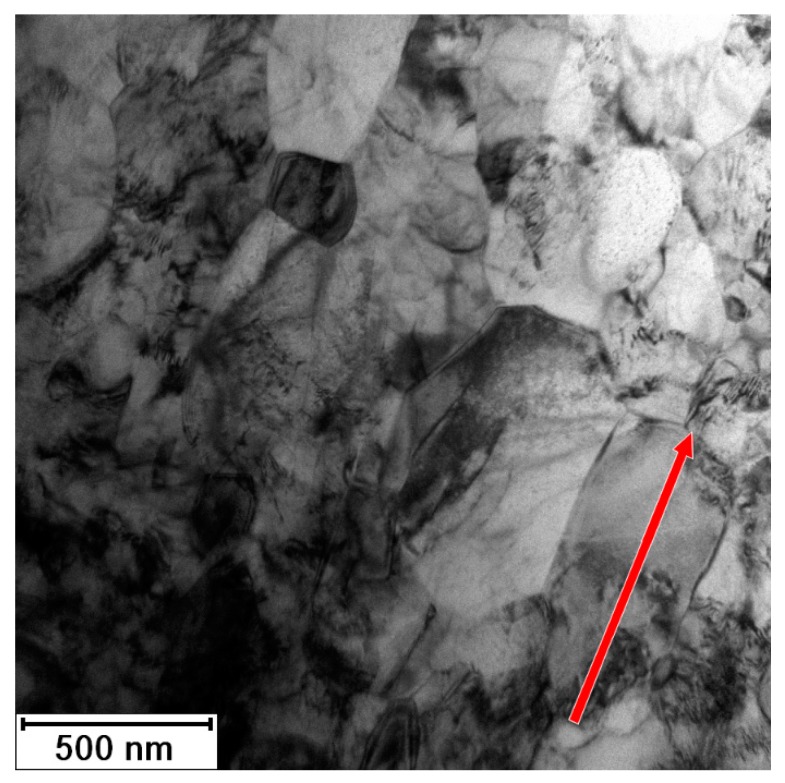
Microstructure of Ti grade 4 after 3 Conform SPD + RS + 450 °C/1 h; arrow shows RS direction (TEM).

The microstructure of the material was significantly changed after annealing at 450 °C for 1 h, as illustrated in the micrograph in Figure 12. The impurities were not detected here. The material is formed of the polyhedral recrystallized grains with last signs of prime preferential orientation. According to XRD (Figure 5), the residual stresses should be released after 1 h of annealing at this temperature. The dark regions (especially noticeable in bottom part of Figure 12) are evidence of some residual stress, but they can be also caused be TEM sample preparation. The grain size is 200–500 nm (minimal Feret’s diameter is 260 ± 130 nm, and maximal Feret’s diameter is 480 ± 250 nm). The size of coherently diffracting domains was estimated to be approximately 200 nm. It means that large grains contain several subgrains, while smaller grains do not. This is an intermediate state of recrystallization, as verified by the hardness measurements presented in Figure 6, where the sample annealed at 450 °C for 1 h is still above the drop in the hardness.

**Figure 13:**
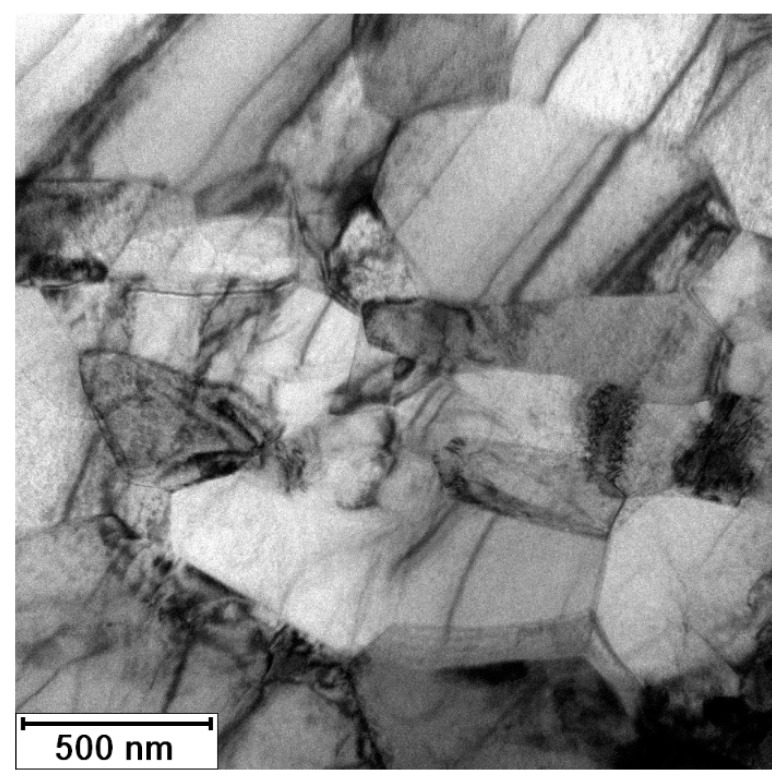
Microstructure of Ti grade 4 after 3 Conform SPD + RS + 450 °C/3 h (TEM).

The microstructure of the sample annealed at 450 °C for 3 h is shown in Figure 13. It contained completely recrystallized polyhedral grains. The average value of the grain size remains almost the same as in the material annealed for 1 h (maximal Feret’s diameter is 530 ± 40 nm, and minimal Feret’s diameter is 300 ± 200 nm).

**Figure 14:**
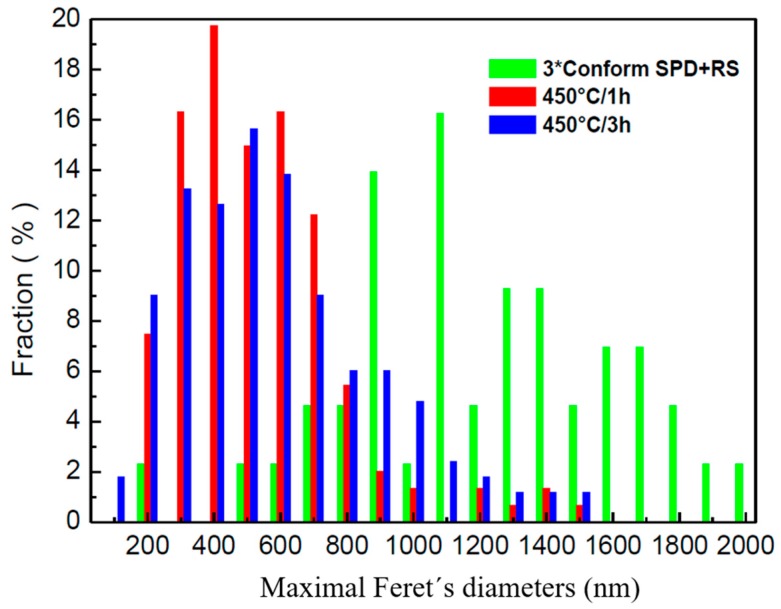
Maximal Feret´s diameters.

**Figure 15:**
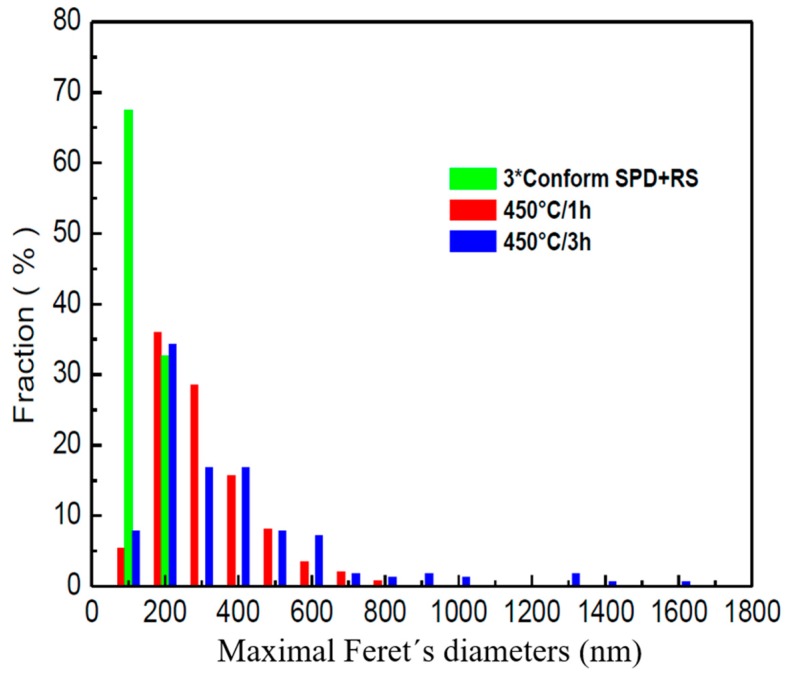
Minimal Feret´s diameters.

A detailed distribution of Feret’s diameters is given in Figure 14 and Figure 15. No subgrains are visible inside the grains. Crystallite size was estimated to be 400 nm, which corresponds to the grain size. Dark lines in Figure 13 might suggest that the stress in the material was probably introduced during preparation of the TEM sample, as they have the same direction independently of the orientation of the grains. The TEM observations are in good agreement with the hardness measurements presented in Figure 7. The decrease of the values of the hardness is not as significant as by annealing at elevated temperatures. 

**Figure 16:**
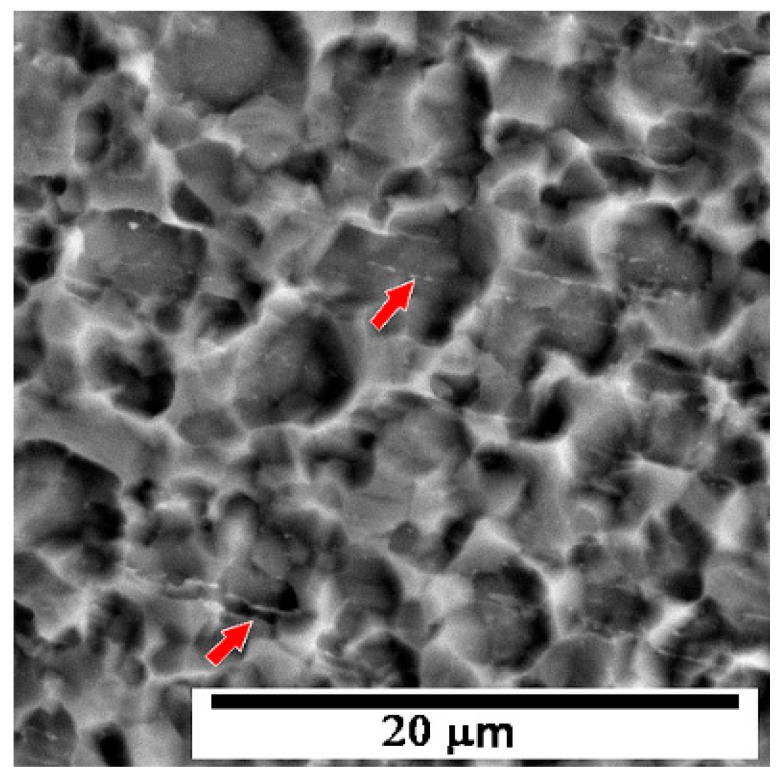
Microstructure of Ti grade 4 after 3 Conform SPD + RS + 525 °C/1 h (SEM).

Annealing at 525 °C for 1 h caused significant grain coarsening. The gain size in this case is 2–7 µm (Figure 16). The grains are too large for both the TEM observation and for the estimation of their size from diffraction patterns. The arrows in Figure 16 show the Fe rich rows that are arranged along processing direction. The distance between individual rows is about 4 µm, which explains why the impurities were not observed in other samples (due to limited field of view in TEM). The significant grain coarsening explains the decrease of the hardness shown in Figure 6.

The evolution of the lattice defects with annealing in the samples after three passes by Conform SPD and rotary swaging was studied by positron annihilation spectroscopy (PAS). The annealing conditions were consistent with the previous measurements: 450 °C/1 h, 450 °C/3 h and 525 °C/1 h.

**Figure 17:**
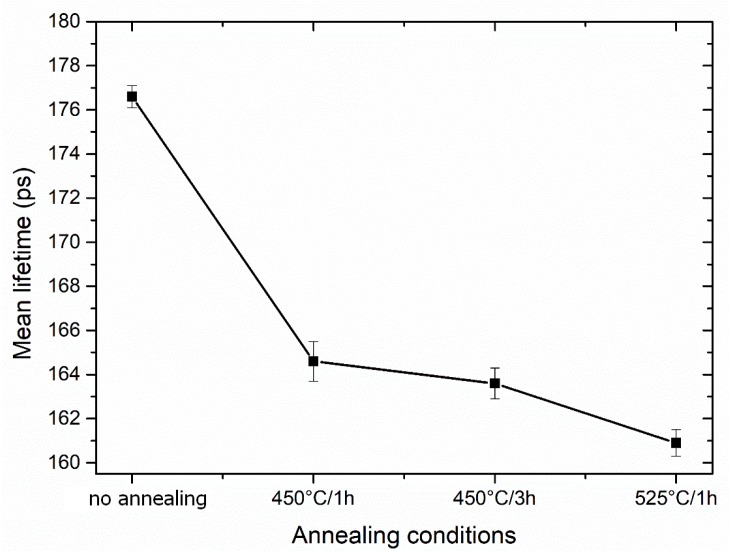
The mean positron lifetime depending on the annealing conditions of the 3 Conform SPD + RS materials.

Figure 17 shows the dependence of the mean positron lifetime on the annealing conditions of the 3 Conform SPD + RS material. In the initial sample, the mean lifetime of positrons is around the values of (176.6 ± 0.5) ps. The calculated bulk lifetime of pure, non-deformed Ti is (144.6 ± 0.5) ps, which is lower compared to the deformed counterpart [17]. The deformation causes a significant increase of the mean lifetime due to presence of the defects introduced by SPD. The mean lifetime abruptly decreased already after a short-time annealing of 450 °C/1 h down to values (164.6 ± 0.9) ps. The continuous decrease can be attributed to the ongoing recovery and recrystallization processes. 

**Figure 18:**
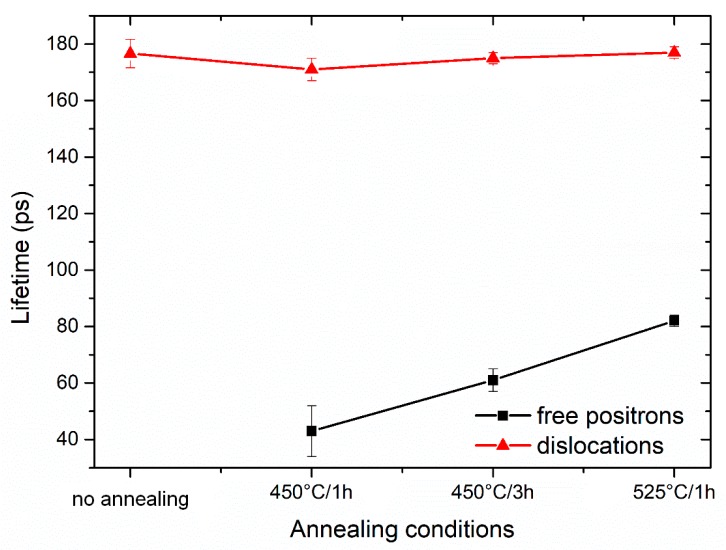
The dependence of positron lifetimes of the individual components resolved in positron lifetime spectra on the annealing conditions.

**Figure 19:**
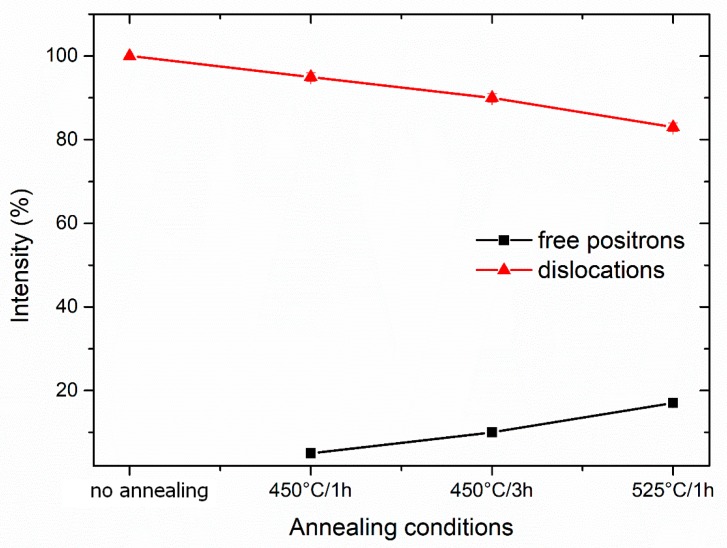
The dependence of intensities of the individual components resolved in positron lifetime spectra on the annealing conditions.

More information can be obtained from the decomposition of positron lifetime spectra into individual components. In Figure 18 and Figure 19, the dependence of the lifetimes and intensities of components resolved in positron lifetime spectra on the annealing conditions is shown, respectively. One can see in Figure 18 and Figure 19 that there are two components in the positron lifetime spectra of the sample studied:

(i) Short-lived component with the lifetime lower than 100 ps represents a contribution from positrons annihilated in the delocalized state, i.e., not trapped at defects. This component was not found in the deformed and non-annealed sample (3 Conform SPD + RS), meaning that the density of defects is so high that virtually all positrons are trapped in the defects’ so-called saturated positron that trapping occurs [18].
(ii) Component with a lifetime ~ 180 ps. This component of the lifetime spectra can be attributed to positrons trapped at the dislocations.

In contrast to other Ti alloys, no other type of defects, such as vacancies, were recognized in the material [19].

The evolution of the defects in 3 Conform SPD + RS sample with annealing is well-characterized by the lifetimes and intensities of each components. As it was mentioned above, in the non-annealed specimen, all positrons are trapped at dislocations, i.e., the positrons do not annihilate as “free positrons”. The lifetime of the positrons after different annealing conditions does not change remarkably, which means that the nature of dislocations remains unchanged during annealing. The component of free positrons with lifetime (43 ± 9) ps appears after annealing at 450 °C for 1 h and can be attributed to partial recovery of defects. The lifetime and also intensity of the free positron component continuously grow with annealing time (450 °C/3 h), while annealing at 525 °C for 1 h causes the most remarkable recovery or possible recrystallization. 

**Figure 20:**
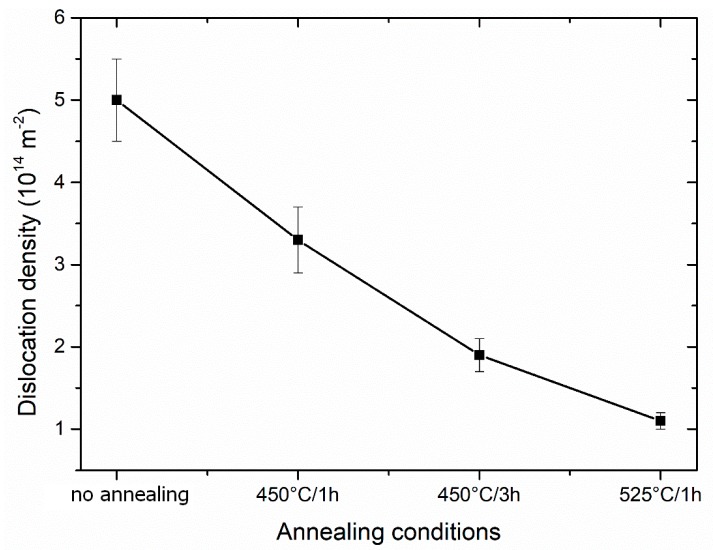
Density of dislocations of Ti grade 4 in the initial state (3 Conform SPD + RS) and after heat treatment (calculated using the STM).

The dislocation density can be estimated from the simple trapping model (STM) [20] if the free positron component is present. The equation used for STM is as follows:

(1) $$\rho_{D}=\frac{I_{D}}{\nu_{D}}\left(\frac{1}{\tau_{f}}-\frac{1}{\tau_{D}}\right)$$

where τ_f_ is the lifetime component of free positrons, τ_D_ is the lifetime component attributed to the positrons trapped at dislocations, ID is the intensity of the positrons trapped at dislocations and νD ≈ 0.5 × 10^−4^ s^−1^m^2^ is the specific positron trapping rate for dislocations [21]. The obtained dislocation density is shown in Figure 20. The saturated trapping at dislocations is marked as dislocation density of (5 ± 0.5) × 10^14^ m^−2^. The density of dislocations continuously decreases with annealing and reaches the lowest values of (1.1 ± 0.1) × 10^14^ m^−2^. 

**Figure 21:**
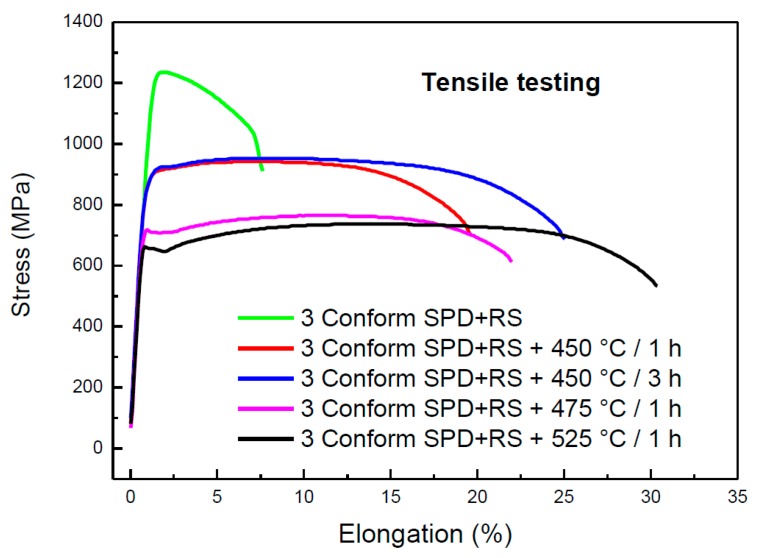
Tensile curves of Ti grade 4 in the initial state (3 Conform SPD + RS) and after heat treatment.

Mechanical properties of selected states of the materials were characterized by tensile testing. Typical curves are shown in Figure 21. The material after severe plastic deformation (3 Conform SPD + RS) is extremely strengthened, and because of this, the shape of the tensile curve differs from the other ones. This material exhibits low elongation to fracture. Annealing at 450 °C/1 h caused a drop of both YS and UTS. The difference between the values of YS and UTS increases is beneficial for potential processing of material, as well as for an increase of ductility. Evidence of slight strengthening during tensile testing is detectable on both curves of material annealed at 450 °C. This effect is pronounced on curves obtained from materials annealed at higher temperatures. Higher temperature of annealing reduces YS, while the ductility rises for all samples annealed for 1 h.

Table 3 summarizes the results of the tensile testing. The average values of YS, UTS and elongation to fracture follow the values of typical curves showed in Figure 21.

The effect of grain size on the value of YS is described by the Hall–Petch Equation:

σ_Y_ = σ_0_ + kd^−1/2^ (2)

where the value for the Hall–Petch slope parameter for titanium is k = 6 MPa mm^1/2^, and the value of σ_0_ as 441 MPa [12]. In case of the material after SPD, the d value should represent subgrain size. For the initial material (3 Conform SPD + RS), the subgrain size was estimated to be 20 nm, which corresponds to σ_Y_ = 1782 MPa. This value is significantly higher than the experimentally obtained value given in Table 3. It is probably caused by preferential orientation of the microstructure and high residual stresses. The same reason is responsible for the discrepancy of theoretical and measured YS values for material annealed at 450 °C for 1 h (σ_Y_ = 931 MPa). For the fully recrystallized materials, the values are in agreement in terms of experimental errors (σ_Y_ = 741 MPa for material with the grain size of 400 nm annealed at 450 °C for 3 h and σ^Y^ = 575 MPa for material with the grain size of 2–7 µm annealed at 525 °C for 1 h). The initial material has mechanical properties comparable to analogous materials [6,12]. Annealing at 450 °C for 3 h preserved high values of YS and UTS accompanied with expressive soar of ductility, and this heat treatment seems to be the optimal one.

## 4. Conclusions

Ti grade 4 was studied in as-prepared plastically deformed state (three passes by Conform SPD and subsequent rotary swaging) and after different regimes of the heat treatment. In situ electrical resistance and in situ XRD proved substantial microstructural changes in the material at the temperature of 450 °C. These changes can be attributed to the relaxation of internal stresses at higher temperatures, followed by recrystallization and grain coarsening. These microstructural changes influenced macroscopic properties—hardness, yield stress, ultimate tensile stress and ductility. This study showed that it is also possible to tune mechanical properties of this material by appropriate annealing. In this respect, annealing at 450 °C for 3 h can be the most suitable regime recommended, which leads to a release of internal stresses and a change of grain shape from elongated to polyhedral with preservation of small grain size. After this heat treatment, the yield stress and ultimate tensile stress achieve the values of 871 and 950 MPa, respectively. The material also reaches a high elongation of 25.1% after this heat treatment.

## Figures and Tables

**Table 1:** Properties of Ti grade 4, N-number of ECAP passes, T-processing temperature, d-grain size, YS-yield stress, UTS-ultimate tensile stress, A-elongation to fracture, dr means drawing and RS means rotary swaging.

N	T (°C)	d (nm)	YS (MPa)	UTS (MPa)	A (%)	Ref.
0	-	25000	625	760	29.3	[6]
1	200	-	853	875	25.7	[6]
2	200	-	869	884	13.7	[6]
4	200	230	921	962	16.2	[6]
6	200	174–220	973	1020	13.7	[6]
8	200	195–230	966	1030	13.4	[6]
10	200	-	950	1020	14.3	[6]
6 + dr	200	150	1190	1230	11	[6]
0	-	12500	560	650	24	[7]
1	220	300–400	707	750	22	[7]
2	220	300–400	740	760	22	[7]
3	220	400–450	756	773	23	[7]
3	200		557	636	24	[5]
3 + RS	200		1136	1142	3.5	[5]
0		20000	483	550	15	[12]
4 + dr	350	200	1110	1250	13	[12]
4 + dr + 450 °C/1 h	350	200	1200	1430	12	[12]

**Table 2:** Mechanical properties of Ti grade 4, YS-yield stress, UTS-ultimate tensile stress and A-elongation to fracture [13].

State	YS (MPa)	UTS (MPa)	A (%)
As-received	362 ± 3	450 ± 5	48 ± 2
4P (ECAP)	462 ± 2	627 ± 1	29 ± 2
4P(ECAP) + A1(300 °C/15 min)	561 ± 3	663 ± 4	29 ± 1
4P(ECAP) + A2(300 °C/30 min)	558 ± 2	651 ± 4	30 ± 2
4P(ECAP) + A3(300 °C/60 min)	542 ± 3	624 ± 7	34 ± 1

**Table 3:** Mechanical properties of Ti grade 4 after 3 passes in Conform SPD, subsequent rotary swaging and heat treatment, YS—yield stress, UTS—ultimate tensile stress and A—elongation to fracture.

State	YS (MPa)	UTS (MPa)	A (%)
3 Conform SPD + RS	1205 ± 7	1224 ± 10	7.8 ± 0.9
3 Conform SPD + RS + 450 °C/1 h	848 ± 17	925 ± 11	17.3 ± 2.2
3 Conform SPD + RS + 450 °C/3 h	871 ± 22	950 ± 11	25.1 ± 1.2
3 Conform SPD + RS + 475 °C/1 h	698 ± 22	757 ± 20	23.4 ± 1.8
3 Conform SPD + RS + 525 °C/1 h	645 ± 14	734 ± 7	32.7 ± 2.7
